# Vagal Nerve Biofeedback Intervention for Improving Health Outcomes Among Ukrainian Forced Migrants: A Proof-of-Concept Study

**DOI:** 10.3390/ijerph22040515

**Published:** 2025-03-28

**Authors:** Yori Gidron, Einav Levy, Chen Hanna Ryder, Sharon Shaul, Rita Sirota, Drorit Atias

**Affiliations:** 1 Faculty of Welfare and Health Sciences, University of Haifa, Haifa 3103301, Israel; ygidron@univ.haifa.ac.il; 2 Department of Social Work, Tel-Hai College, Qiryat Shmona 1220800, Israel; 3 Research Center for Innovation in Social Work, Tel-Hai College, Qiryat Shmona 1220800, Israel; 4 Brain & Behavior Research Institute, Western Galilee Academic College, Akko 2412101, Israel; chenr@wgalil.ac.il; 5 Clalit Health Services, Tel Aviv 6250769, Israel; 6 Department of Cardiology, Carmel Hospital, Haifa 3103301, Israel; 7 Faculty of Medicine, The Hebrew University, Jerusalem 9112102, Israel; drorit.attias@mail.huji.ac.il

**Keywords:** Ukraine conflict, health conditions, vagal nerve, paced breathing, humanitarian action

## Abstract

Background: The ongoing conflict in Ukraine has forced numerous migrants into neighboring countries, many suffering from pre-existing or newly acquired physical and mental health conditions. Addressing these complex challenges in humanitarian settings requires innovative, evidence-based interventions that are cost-effective and easy to administer. Drawing upon research highlighting the vagus nerve’s role in regulating well-being, we hypothesized that vagal nerve activation could offer a promising therapeutic approach. Method: We conducted a proof-of-concept study in which 21 Ukrainian forced migrants were trained in a biofeedback-guided paced breathing intervention designed to stimulate the vagus nerve and promote self-regulation of stress response systems. Changes in pain perception, perceived stress, blood pressure, and heart rate were assessed before and after the vagal breathing intervention using a *t*-test. Correlations were examined at baseline. Results: Statistically significant improvements were observed in all measures except systolic blood pressure, providing preliminary evidence for the efficacy of vagal nerve activation in alleviating stress-related health symptoms. Conclusions: This study demonstrates the feasibility and therapeutic potential of a vagal nerve-activating intervention in a humanitarian setting. These findings warrant replication in larger, controlled trials. If substantiated, this low-cost, scalable intervention could help mitigate health burdens among forced migrant populations worldwide.

## 1. Introduction

The Russian invasion of Ukraine on the 24th of February 2022 marked the beginning of the fastest and largest forced migration movement in Europe since the Second World War. As of June 2022, the ongoing conflict has displaced over 4.2 million Ukrainians, compelling them to seek refuge in neighboring countries [1]. Poland, Slovakia, Hungary, and Romania have emerged as the primary destinations for these forced migrants, with the European Union (EU) granting them official recognition as war refugees [2]. Notably, Poland has received the largest influx of people of concern (POC) crossing the border, owing to its 500 km shared border with Ukraine, close familial ties, and linguistic familiarity [3]. The demographic composition of this refugee population is heavily skewed, with women and children accounting for 90% of the POC, as Ukrainian legislation mandates military service for men aged 18–60 [4]. Upon crossing the borders, these forced migrants are provided with basic humanitarian support, including food, clothing, shelter, medical care, and psychosocial assistance, in specially established centers and transit camps.

In contexts where migrants have undergone a treacherous journey, the profile of medical conditions they present with is shaped by a complex interplay of factors. These include pre-existing health risks and conditions in their country of origin, exposure to war-related injuries and atrocities, and health issues acquired during their perilous voyage [5]. Additionally, sociodemographic variables such as age, gender, and lifestyle contribute to the diverse health profiles observed among forced migrant populations. For instance, a study conducted among 1550 Syrian refugees residing in forced migrants’ camps in Jordan found that the leading chronic diseases were hypertension (9.7%), arthritis (6.8%), diabetes (5.3%), cardiovascular diseases (3.7%), and respiratory diseases (3.1%) [6]. Similarly, research on the morbidity of forced migrants along the Balkan route (Greece, North Macedonia, Serbia, and Croatia) who fled the Middle East due to the Syrian conflict and its regional repercussions indicates that infectious diseases are the most prevalent health concern among these refugees [7].

Alongside the physical toll of forced migration, such experiences can lead to a wide range of mental health sequelae, including psychopathology. Numerous studies have documented elevated rates of psychological disorders, particularly post-traumatic stress disorder (PTSD), among refugee and forced migrant populations [8,9]. For example, a study of Syrian refugees in Turkey revealed a PTSD prevalence of 33.5% [10], while another investigation of 781 Syrian refugees found that 83.4% met the criteria for probable PTSD and 37.4% for probable depression [11].

These high rates of psychopathology have been consistently observed across various settings and age groups. A study of 38 Yazidi children found that all participants exhibited psychiatric symptoms, with 71% reporting sleep disturbances, 36.8% screening positive for depression, and 10.5% for PTSD [12]. Similarly, a study of Yazidi children revealed that 36.4% met criteria for PTSD, 32.7% for depression, and 7.3% for anxiety [13]. Among 847 Somali refugees living in Southeast Ethiopia, 38.3% were found to be depressed [14]. This pattern was also observed in a study of 200 male forced migrants from West Sub-Saharan Africa, which reported a PTSD prevalence of 12% [15].

It is important to note that the aforementioned studies were conducted among refugees and forced migrants who were primarily located in close proximity to their country of origin. The variation in prevalence rates across studies may be attributed to differences in sampling methods, assessment tools, and the duration of time elapsed since exposure to war-related trauma or atrocities. While there is a growing body of evidence supporting the effectiveness of mental health and psychosocial support (MHPSS) interventions, the tools currently in use often lack cultural adaptation, necessitating a critical examination of these findings [16].

### 1.1. The Case of Ukraine

Prior to the outbreak of war, the five leading causes of mortality in Ukraine, accounting for 84% of all deaths, were cancer, chronic obstructive pulmonary disease (COPD), cardiovascular disease, diabetes, and mental diseases [17]. Hypertension affected 35% of the Ukrainian population, with 85% of cases being uncontrolled [17], highlighting the urgent need for medical management of this serious health risk. Cancer was responsible for 13% of deaths in Ukraine, with colorectal, breast, and lung cancer being the most common types. Diabetes had a prevalence of approximately 7% among Ukrainians [17], although the true figure is likely higher, as a quarter of the population had never undergone blood tests. A review of studies found the following health problems to prevail among Ukrainians: COVID-19 infections, tuberculosis with drug resistance, high drug resistance, and difficulties with national vaccination [18]. In a study on Ukrainians hospitalized in Poland, the most common health problems before Feb 2022 included injuries, childbirth, and puerperium, among others. After this period, these problems remained prevalent, but abnormal lab findings and infections increased as well [19].

Regarding mental health, 12.4% of Ukrainians had depression prior to the war, but only 3.2% had ever received treatment for this condition [17]. The psychological impact of the conflict is expected to compound this burden, with increased rates of anxiety, PTSD, and grief, further straining the already limited healthcare resources [20,21,22].

Following the outbreak of the war with Russia, several humanitarian agencies have been providing support to forced migrants in the surrounding countries, as mentioned earlier. The most common health conditions encountered include acute illnesses (e.g., upper respiratory infections, injuries) and chronic diseases (e.g., hypertension, diabetes). These conditions are typically managed with basic medical care, such as medication (e.g., beta-blockers, antibiotics) and wound dressings.

However, this approach to treatment is not without challenges. First, the lack of resources and infrastructure in humanitarian settings precludes the use of advanced diagnostic measures (e.g., echocardiography, X-rays, blood tests, and brain imaging) to assess clinical outcomes. Second, the transient nature of the POC population, as they continue their onward journey, hinders long-term treatment, therapeutic effectiveness evaluation, monitoring of biomarker changes (e.g., liver function, insulin levels), and management of medication side effects. Finally, the limited financial capacity of humanitarian organizations and the Ukrainian system constrains their ability to provide comprehensive mental and physical healthcare and evaluation [23].

Consequently, there is an urgent need to develop and implement a logistically simple, cost-effective intervention that can address many of the aforementioned health conditions and their underlying pathology, ideally without side effects. Moreover, it is crucial that patients can self-administer the treatment, reducing their dependence on a constantly changing roster of healthcare providers in the context of migration. Finally, it is of high importance to identify a protective factor that is common to many diseases, and it is easy to monitor its activity and activate it.

### 1.2. A Common Denominator of These Health Problems: The Vagal Nerve

The vagus nerve emerges as a common protective neurobiological factor that is correlated with the health conditions mentioned above via plausible evidence-based biological mechanisms.

The vagus nerve, the tenth cranial nerve, originates in the brainstem and descends to the viscera, innervating most organs. Its activity can be measured non-invasively through heart rate variability (HRV), reflecting fluctuations in the intervals between normal heartbeats. HRV has a strong correlation with actual vagal nerve activity (r = 0.88) [24]. Crucially, low HRV is predictive of the onset of hypertension [25], and a review of 25 studies found that HRV is consistently lower in individuals with diabetes mellitus [26]. High HRV is associated with a lower risk of developing myocardial infarction (MI) [27] and a four-fold increase in post-MI survival, as demonstrated by a review of 21 studies [28]. Similarly, in cancer, high HRV is linked to better prognosis and survival, independent of confounding factors [29,30].

HRV is negatively correlated with inflammation, as found in a meta-analysis of 51 studies [31]. In a review of 25 studies, HRV was found to be already apparent in pre-diabetes [32]. Finally, high HRV also predicts a lower risk of heart failure in women [33]. In cardiac patients, high HRV predicts a better prognosis [34].

With regard to mental health, a review of 36 studies found that HRV is reduced in generalized anxiety disorder, panic disorder, and PTSD [35]. Furthermore, a review of 21 studies revealed that HRV is also lower in depression [36]. HRV has also been found to be positively correlated with prefrontal cortical activity [37].

The biological mechanisms linking vagal activity to these health conditions can be understood through the lens of neuroimmunology. Inflammation is a key contributing factor in many of the aforementioned health problems [38]. The vagus nerve plays a pivotal role in communicating peripheral inflammation to the brain [39] and subsequently inhibiting it through two pathways. First, vagal activation stimulates the hypothalamic–pituitary–adrenal axis, resulting in the release of cortisol, which suppresses inflammation [40]. Second, descending vagal afferents reach the celiac ganglion, where they transition to a sympathetic branch that innervates the spleen. There, beta-adrenergic receptors on a subset of resident T cells receive the sympathetic signal and, in response, secrete acetylcholine. Acetylcholine then binds to alpha-7 nicotinic acetylcholine receptors on splenic macrophages, which inhibits the production of pro-inflammatory cytokines [41,42].

Electrical stimulation of the vagus nerve has been shown to reduce infarct size [43], alleviate chronic pain [44], and enhance antiviral immunity, including increased NK cell and CD8 T-cell counts [45]. Importantly, the vagus nerve can be non-invasively activated through HRV biofeedback (HRV-B).

Taking it to a higher level of the brain, HRV is positively correlated with more connectivity between the prefrontal cortex and the amygdala [46]. Furthermore, similar connectivity between the ventromedial prefrontal cortex and the amygdala was found to be inversely related to both anxiety and inflammation [47]. Thus, theoretically, by activating the vagus, such connectivity may increase, and this would serve to reduce anxiety and inflammation in forced migrants.

In HRV-B, patients learn to perform paced breathing while receiving real-time feedback on their HRV level via a mobile phone screen. Within seconds, they learn to increase their own HRV, which is empowering and fosters a sense of control over their health. This sense of control is particularly crucial in the context of crisis, uncertainty, and helplessness. Furthermore, the simplicity of this approach and its low cost make it highly relevant and attractive in the context of treating patients with little resources. Several reviews found that HRV biofeedback improves emotional and physical health [48,49,50]. Moreover, HRV biofeedback and vagal breathing have been shown to reduce inflammation, pain, and anxiety (e.g., [51,52,53]). However, to the best of our knowledge, the effects of HRV-B have not been systematically examined in humanitarian contexts or across the multiple medical conditions discussed above.

The following study represents a proof-of-concept intervention within the context of the Ukrainian humanitarian crisis. Due to the ethical imperative and medical necessity of providing care to all those in need, this study was not designed as a formal randomized controlled trial. Nevertheless, we obtained ethical approval from the [institution name] (#BLIND TO REVIEWERS, Ref number 9-6/2022) to collect the reported data, which were gathered for medical purposes and shared with patients as part of standard good clinical practice (GCP). Informed consent was obtained from all participants, with forms provided in their native language outlining this study’s objectives, applications, and contact information for the principal investigator.

## 2. Methods

The worldwide disaster relief non-governmental organization (NGO) NATAN, originally based in Israel, has been operating in Poland since March 2022, providing emergency medical and psychosocial support through a clinic located in a transit camp in Przemysl.

During the period in which the intervention took place, the transit center housed approximately 4000 people of concern (POC) at any given time, with an average length of stay of 1.5 days. The clinic, staffed by physicians, nurses, and social workers, many of whom were proficient in Russian or Ukrainian, offered round-the-clock primary care. The standard operating procedure involved patient registration, collection of demographic information (age, gender) and medical history, diagnostic assessment, and provision of treatment based on clinical judgment and available resources.

The primary health concerns addressed at the NATAN clinic encompassed a range of acute and chronic conditions, including infectious diseases (particularly gastroenteritis and upper respiratory tract infections [URTI]), minor traumatic injuries and wounds, musculoskeletal complaints, headaches, and psychopathology (e.g., anxiety, depression, and sleep disturbances). Additionally, the clinic managed cardiovascular issues, such as acute exacerbations of ischemic heart disease, congestive heart failure, and asthma.

Treatment modalities included oral medications such as paracetamol, non-steroidal anti-inflammatory drugs (NSAIDs), antibiotics, and pharmacotherapies for the symptomatic relief of cold, flu, diarrhea, nausea, and vomiting, as well as antidepressants and anxiolytics. In emergency situations, intravenous medications, including Fusid and steroids, and inhaled therapies, such as Ventolin and Aerovent, were administered. The clinic also provided ongoing care and follow-up for chronic conditions, including hypertension, diabetes, arthritis, hypothyroidism, epilepsy, malignancies, and gastrointestinal disorders such as peptic ulcers and heartburn.

## 3. Patients

The sample comprised 21 Ukrainian patients who sought care at the NATAN humanitarian organization’s clinic in Przemysl. These were referred to the HRV-B intervention only if their health condition was related to low vagal activity (e.g., pain, anxiety, hypertension, cardiac disease). Importantly, this was not planned to be a research but rather an intervention in which we measured the outcomes out of good clinical practice. Therefore, we included only 21 patients, reflecting a proof of concept. Table 1 presents the prevalence of their primary health concerns. Participants ranged in age from 10 to 80 years, with a mean (SD) age of 41.33 (20.01) years. The sample included 9 males (42.9%) and 12 females (57.1%).

## 4. Measures

Data collected included patients’ age, gender, and the primary health complaint that prompted their clinic visit. Additionally, perceived stress and current subjective pain levels were assessed using a 0–10 rating scale. Blood pressure, including systolic blood pressure (SBP) and diastolic blood pressure (DBP), was measured using a Life Ltd. electronic blood pressure monitor (model number KD-558).

To measure heart rate variability (HRV), we employed the I-feelwell photoplethysmograph (PPG) device, which records HRV, heart rate, and oxygen saturation via a sensor attached to the left index finger. This device provides two time-domain HRV parameters: the standard deviation of normal-to-normal (SDNN) interbeat intervals and the root mean square of successive differences (RMSSD) between adjacent normal-to-normal intervals. A recent study found that HRV measured using this device significantly predicted episodes of Crohn’s disease in children [54]. A control group was unsuitable for this intervention for two reasons: (1) This was a humanitarian crisis where all patients deserved the best available treatment, and HRV-B has proven health benefits [50]; (2) Comparing this cohort to the other patients was not suitable since the other patients’ health conditions were unrelated to low HRV.

Measures were performed before and immediately after the HRV-B without follow-ups. 

## 5. Interventions

The intervention consisted of an educational component and training in a 3-minute paced vagal breathing technique. This was a single-session intervention due to the nature of the context in which migrants were in transit. In such a chaotic context, it was challenging to monitor and provide additional treatment. This was additional to medical care without any other elements. First, we used clear, laminated visual aids to explain to each patient how vagal nerve activation relates to their specific health condition. These materials included a diagram illustrating the connection between their condition and the vagus nerve, along with a summary of research demonstrating the benefits of vagal breathing for that particular health concern. Next, we provided instructions on the vagal breathing technique and gave patients a laminated summary card in Russian (the language spoken by most or all Ukrainian participants), which was also used by our healthcare staff. This laminated information card was provided to encourage patients to continue practicing vagal breathing during their migration journey when physicians are not available. Moreover, this empowered patients to perform self-health care. Patients then practiced the 3-minute deep, paced breathing exercise as follows: inhale through the nose for a count of 1–5, hold the breath for a count of 1–2, and exhale through pursed lips for a count of 1–5. This breathing technique has been shown to increase HRV and improve decision-making [55].

## 6. Procedure

All patients underwent a baseline assessment of perceived pain and stress, SBP, DBP, and HRV during a 30 s resting period. They then received the educational explanation and performed the 3-minute paced breathing exercise. Immediately following the intervention, perceived pain and stress, SBP, DBP, and HRV were reassessed during another 30 s resting period, during which time patients were instructed to breathe normally.

## 7. Statistical Analysis

We report descriptive statistics, including means for continuous variables and percentages for categorical variables. The effects of vagal breathing on all outcome measures were evaluated using paired *t*-tests. Additionally, we examined correlations between the baseline measures to validate the assessments and gain further insight into the relationships among the collected variables.

## 8. Results

Regarding the distribution of categorical variables, 42.9% of the participants were male, and 57.1% were female. Nearly half of the sample (47.6%) had a chronic systemic disease, including hypertension (n = 8), epilepsy (n = 1), or peripheral numbness (n = 1). Chronic pain was reported by 28.6% of the participants, while 23.8% experienced anxiety or severe stress. Table 1 presents the means (SD) of all continuous variables before and after the vagal breathing intervention.

Most parameters were normally distributed at baseline; thus, we did not perform any adjustments to normalize the data except for SDNN. We used paired *t*-tests for DBP and tress, and for all the others, we used the Wilcoxon rank test for paired samples. All outcome measures, with the exception of SBP, showed significant improvements following the vagal breathing exercise. Specifically, DBP (t(19) = 1.96, *p* < 0.05, one-tailed), pain (w = 0, *p* < 0.01), and perceived stress (t(12) = 4.77, *p* < 0.001) were significantly reduced post-intervention. In contrast, RMSSD (w = 147, *p* < 0.005) and SDNN (w= 160.5, *p* < 0.0005) significantly increased following the vagal breathing exercise. Due to the small N, we did not control for the effects of confounders that were available to us (Type of disease).

Table 2 presents the correlations between the outcome variables at baseline. We focused on the SDNN HRV parameter, which was highly correlated with RMSSD (r = 0.90, *p* < 0.01) and demonstrated more consistent associations with the other variables. Both SBP and DBP exhibited strong, positive, and significant correlations with perceived stress. SDNN was negatively and significantly correlated with both pain and perceived stress. Interestingly, pain and perceived stress were not significantly correlated with each other.

## 9. Discussion

This pioneering project represents the first application of neuroimmunology and psycho-physiological principles in a humanitarian context. Specifically, we tested the effects of an evidence-based heart rate variability biofeedback (HRV-B) intervention on multiple health conditions among Ukrainian forced migrants.

Our findings demonstrate significant improvements in five out of six outcome measures, encompassing both objective and subjective indices. In particular, diastolic blood pressure (DBP), pain, and perceived stress were reduced, while two HRV parameters were increased significantly following the intervention.

The observed correlations between the vagal index and other outcomes, as well as the effects of HRV-B on medical and subjective measures, underscore the integrative and comprehensive roles of the vagus nerve in health. This notion is supported by the neurovisceral integration model [56] and a neuroimmunological model of chronic diseases [57]. The neurovisceral integration model highlights the central role of the vagus nerve, as indexed by HRV, in mediating the relationship between frontal brain activity and the regulation of cardiac function. The neuroimmunological model of chronic diseases synthesizes evidence linking vagal activity to healthier behaviors (e.g., reduced smoking), the predictive value of its marker (HRV) for lower risk and better survival in chronic diseases, and the regulation of biological disease mechanisms (e.g., inflammation).

Our results align with the findings of Lehrer et al. [48] and Gitler et al. [50], who reviewed the effects of HRV-B on various health conditions, including hypertension, chronic pain, and heart disease. Specifically, our observations are consistent with studies demonstrating that HRV-B reduces blood pressure and pain [51,52]. Furthermore, our findings concur with a meta-analysis of 24 studies showing that HRV biofeedback reduces anxiety [58].

## 10. Conclusions and Implications to Policy

This proof-of-concept intervention study supports the acceptability and effectiveness of self-activation in humanitarian contexts. This preliminary study needs to be replicated in a larger sample with a control group, respecting the unique ethical challenges of humanitarian contexts. If replicated, this novel psycho-physiological approach may offer a simple but not simplistic intervention for a wide range of health problems.

### 10.1. Limitations

The present project was, first of all, a humanitarian project, and as such, it naturally has several limitations. First, the sample size was small and heterogeneous, and there was no control group. However, given that this initiative was conducted during a humanitarian crisis, it was not feasible to design a controlled study while at the same time adhering to good clinical practice principles. Patients were provided only one HRV-B breathing session, while most other studies included 5–8 weeks [59]. This was impossible within the humanitarian context in which we were working. In addition, we did not control the effects of confounders such as sleep quality, physical activity, diet, etc., since this information was not available to us. Moreover, the primary purpose of data collection was to provide patients with feedback on their HRV-B performance rather than to conduct a formal study. Finally, though a laminated information card was provided to patients so they could continue practicing vagal breathing during their migration journey, the long-term effects of this intervention remain unknown. Future studies should aim to conduct follow-ups to monitor the independent performance of vagal breathing and to measure HRV.

### 10.2. Strengths of This Study

Despite these limitations, the intervention is evidence-based, drawing upon extensive findings from neuroimmunology and health psychology research. Moreover, it is simple yet effective, inexpensive, and without any known adverse side effects. To the best of our knowledge, this approach has seldom been applied in humanitarian contexts.

### 10.3. Implications for Policy

Humanitarian crises present a wide range of health challenges for affected populations, including distress related to displacement, witnessing the death and injury of loved ones, personal injury, and lack of treatment for pre-existing health conditions. Measuring HRV and performing vagal breathing with biofeedback are inexpensive interventions that can address many of these health concerns and can be easily taught to staff and patients. Furthermore, patients can practice the technique independently, without relying on the healthcare system, thereby promoting a sense of empowerment. People of concern can engage in HRV-B while on the move and at any given time and place.

If replicated, this intervention could be widely disseminated and easily adopted in multiple humanitarian settings worldwide. The potential impact of such an intervention is substantial, as it may improve the quality of life and health outcomes for a large number of individuals affected by humanitarian crises. Health professionals must be educated about the protective roles of the vagal nerve in fatal and chronic diseases and how to monitor and activate it at a minimum cost. These could be implemented on a routine basis in global southern countries toward early detection, prevention, and possible treatment of NCD for the benefit of global health.

## Figures and Tables

**Table 1:** Mean and Standard deviation of main outcome variables.

	Pre		Post	
Variable	Mean	SD	Mean	SD
SBP	148.6	22.5	143.6	22.1
DBP	90.5	12.6	86.5 *#	12.3
RMSSD	37.7	27.5	55.3 *	49.7
SDNN	48.4	39.2	73.1 ***	48.3
PAIN	3.9	2.4	2.7 *	2.4
STRESS	5.8	2.4	3.1 ***	2.2

SBP = Systolic blood pressure; DBP = Diastolic blood pressure; RMSSD = Route mean square of successive differences; SDNN = Standard deviation of normal to normal intervals; # *p* < 0.05 (one-tailed); * *p* < 0.05; *** *p* < 0.001.

**Table 2:** Correlations between outcome variables.

	SBP	DBP	SDNN	Pain	Stress
DBP	0.52 **	1	−0.25	0.18	0.55 *
SDNN	−0.29	−0.25	1	−0.69 *	−0.77 **
Pain	0.42	0.18	−0.69 *	1	0.28
Stress	0.81 **	0.55 *	−0.77 *	0.28	1

* *p* < 0.05; ** *p* < 0.01.

## Data Availability

The data that support the findings of this study are available from the corresponding author, but restrictions apply to the availability of these data, which were used under license for the current study and so are not publicly available. Data are, however, available from the authors upon reasonable request and with permission of the corresponding author.

## Abbreviations

COPD	Chronic Obstructive Pulmonary Disease
DBP	Diastolic Blood Pressure
EU	European Union
GI	Gastrointestinal
HRV	Heart Rate Variability
MHPSS	Mental Health and Psychosocial Support
MI	Myocardial Infarction
NSAIDs	Non-Steroidal Anti-Inflammatory Drugs
POC	People of Concern
PPG	Photoplethysmography
PTSD	Post-Traumatic Stress Disorder
SBP	Systolic Blood Pressure
SDNN	Standard Deviation of Normal-to-Normal Intervals
RMSSD	Root Mean Square of Successive Differences
UNHCR	United Nations High Commissioner for Refugees
URTI	Upper Respiratory Tract Infections
